# Progression of liver cirrhosis to HCC: an application of hidden Markov model

**DOI:** 10.1186/1471-2288-11-38

**Published:** 2011-04-04

**Authors:** Nicola Bartolomeo, Paolo Trerotoli, Gabriella Serio

**Affiliations:** 1 Department of Biomedical Science and Human Oncology, Chair of Medical Statistics, University of Bari, Bari, Italy

## Abstract

**Background:**

Health service databases of administrative type can be a useful tool for the study of progression of a disease, but the data reported in such sources could be affected by misclassifications of some patients' real disease states at the time. Aim of this work was to estimate the transition probabilities through the different degenerative phases of liver cirrhosis using health service databases.

**Methods:**

We employed a hidden Markov model to determine the transition probabilities between two states, and of misclassification. The covariates inserted in the model were sex, age, the presence of comorbidities correlated with alcohol abuse, the presence of diagnosis codes indicating hepatitis C virus infection, and the Charlson Index. The analysis was conducted in patients presumed to have suffered the onset of cirrhosis in 2000, observing the disease evolution and, if applicable, death up to the end of the year 2006.

**Results:**

The incidence of hepatocellular carcinoma (HCC) in cirrhotic patients was 1.5% per year. The probability of developing HCC is higher in males (OR = 2.217) and patients over 65 (OR = 1.547); over 65-year-olds have a greater probability of death both while still suffering from cirrhosis (OR = 2.379) and if they have developed HCC (OR = 1.410). A more severe casemix affects the transition from HCC to death (OR = 1.714). The probability of misclassifying subjects with HCC as exclusively affected by liver cirrhosis is 14.08%.

**Conclusions:**

The hidden Markov model allowing for misclassification is well suited to analyses of health service databases, since it is able to capture bias due to the fact that the quality and accuracy of the available information are not always optimal. The probability of evolution of a cirrhotic subject to HCC depends on sex and age class, while hepatitis C virus infection and comorbidities correlated with alcohol abuse do not seem to have an influence.

## Background

The evolution of chronic degenerative disease is characterized by progression through intermediate states to advanced disease and death. For these diseases, survival analysis must take into account the various transitions from one state to the next, as well as a series of prognostic variables that can have an influence on each event including death. For example, liver cirrhosis can evolve to hepatocellular carcinoma, and the presence of comorbidities, exposure to hepatitis B or C virus, as well as alcohol consumption and age, can influence the terminal event. In fact, liver cirrhosis is well known to consist of a diffuse alteration of the liver structure resulting from protracted processes of liver inflammation and necrosis of different natures. The main causes of liver cirrhosis are chronic viral hepatitis B or C and the consumption of alcohol. In particular, alcohol abuse can halve the time of onset of cirrhosis in a patient already affected by chronic viral hepatitis (from about 20-30 years to 10-15 years). Hepatocellular carcinoma occurs at a rate of 1% to 4% per year after cirrhosis is established [1] and cirrhosis underlies HCC in approximately 80%-90% of cases worldwide [2]. Stochastic multistate or competing models, like Markov chains, are those best suited to the analysis of such phenomena [3-6]. It would, of course, be too long and costly to program clinical studies, or indeed prospective and follow-up trials, to study the natural history of chronic degenerative diseases in order to be able to apply multistate models correctly. In fact, in patients affected by these diseases passage from one state to the next often occurs after fairly long intervals.

Retrospective studies, based on the use of health service databases of administrative type (HSDBA), can be a valid alternative, despite the limits posed by the fact that the quality and accuracy of the available information are not always optimal. For example, using the hospital discharge sheets (HDS) database, patients can be followed from the probable diagnosis of onset of the disease through the subsequent worsening states. Moreover, by means of linkage with the death certificates database (DCDB), it is ultimately possible to trace the cause of death, if applicable.

A common problem when applying Markov models to HSDBA is that assessment of the disease state of an individual can be subject to classification errors. A similar classification problem frequently occurs in research in the field of social sciences, where wide use is made of a series of models that take into account latent states [7-9].

Multistate models that exploit the properties of Markov chains offer a useful methodological structure for describing complex time-dependent outcomes [10]. The procedure for estimation of the probability of transition most widely adopted in Markov models is the Cox proportional model. This model can describe survival time in function of a multitude of prognostic factors [11], under the fundamental assumption of the proportionality of hazards, in other words that the examined factors will have a constant impact over time on the risk of death.

Jackson et al. (2003) [12] described a procedure for simultaneously estimating the transition rates and the probabilities of misclassification in a hidden Markov model, supplying software for implementing the multistate hidden Markov model in the R Project programming environment. Aim of the present work is to study the pathway leading subjects affected by liver cirrhosis to develop hepatocellular carcinoma and to death, determining the transition probabilities through the degenerative disease states and verifying whether, apart from being risk factors for the onset of cirrhosis, chronic viral hepatitis B and C infection and alcohol abuse also have a role in the process leading cirrhotic subjects to develop hepatocellular carcinoma and/or to death.

## Methods

Retrospective observational studies can be conducted using the HSDBA, to assess the natural history of the disease in a group of subjects. In fact, the HDS database can be employed to individuate all subjects admitted to hospital one or more times, as well as the duration of the interval between one hospitalization and the next. For each hospitalization, the disease state observed and the covariates of interest can be traced. However. the disease state may be affected by an unknown degree of error, due both to an imperfect diagnosis and to incorrect classification. Moreover, due to the irregular nature of follow-up, observations of the actual time of entry into a disease state are frequently interval censored. The proposed Markov model allows us to take into account the above characteristics.

In a sample of *n *subjects, for the *i*th subject we assume that the following variables are observed at the *j*th visit:

T_ij _chronological time of clinical visit

Y_ij _binary disease outcome measurement

Z_ij _vector of covariates.

The observed disease outcome measures Y_ij _are subject to error. The actual underlying disease status is assumed to be a process evolving in continuous time and is denoted (X(t), t > 0). This process is unobserved or 'hidden', and will be modeled as a continuous time two-state Markov process, where the states are interpreted as the presence or absence of the disease manifestation. Let Y_1_^j ^and T_1_^j ^denote the sequence from 1 to *j *of observed disease states and observation times for an individual *i*. The Markov assumption for the hidden disease process is given by(1)

where the quantity Px_j-1_, x_j _denotes the probability of transition to occupy state x at time T_j _= t_j _given that the process was in state x_j-1 _at t_j-1 _and that the transition probabilities are assumed to be stationary. We also assume that, conditional on the state of the hidden process at time t_j_, an observation Y_j _is independent of all previous observations and the hidden process prior to time t_j_:(2)

When Y is binary, f(y|x) can be interpreted as the probability of correctly or incorrectly classifying the disease state given the true state of the subject. The conditional independence of misclassification probabilities at successive time points is a strong assumption. However, dependence between successive measurements is confounded with dependence between the true disease states. It is not possible to disentangle these two features of the model with misclassified data [13].

Equations (1) and (2) constitute a hidden Markov model.

If the disease status is observed accurately, then X(T_j_) and Y_j _coincide, and the model is reduced to a pure continuous time Markov process.

At any time t and for each pair of states r and s, the transition from one state to the next and the time when this transition occurs are regulated by the transition intensity q_rs_. The transition intensity q_rs _represents the instantaneous risk of moving from state r to state s:(3)

The intensities form a matrix Q whose rows sum to zero, so that the diagonal entries are defined by .

On the basis of the available data it is necessary to estimate the matrix Q of transition intensity according to the method described by Kalbfleisch and Lawless [14], and Kay [15]. In addition, at each level of the model explicative variables can be included and it is possible to use a proportional hazards model to relate transition intensities q_rs_(t) to time t with the covariates z(t),(4)

The new matrix of the transition probabilities P(t), that takes into account any necessary covariates, can be calculated by taking the matrix exponential of the scaled transition intensity matrix (see, for example, Cox and Miller [16]):(5)

The hazards proportionality can be verified using the Schoenfeld residuals of the model [17,18], defined as the value *x_ik _*of the covariate K for individual *i *who actually died at time t_i _minus the expected value, where the expected value is given by and *p*_*i *_is the probability of death of individual *i *at time t_i_. The graph, that represents the trend of the Schoenfeld residuals calculated for each individual and each covariate, can be used to directly visualize the hazards ratio [17]. Assuming proportionality of the hazards, the Schoenfeld residuals are independent of time. The presence of a linear relationship with time, an index of non proportionality, can be tested by performing a simple linear regression and a trend test: a slope significantly different from zero would be evidence against proportionality and an increasing (decreasing) trend would indicate an increasing (decreasing) hazards ratio over time.

**Figure 1:**
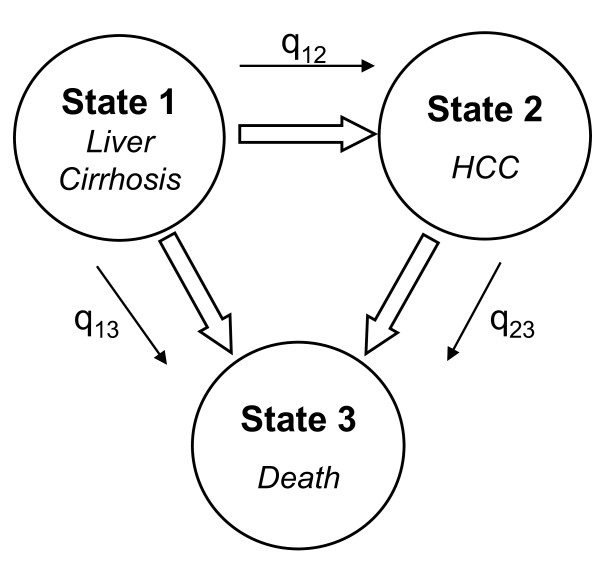
**Three-state hidden Markov model**.

In our model the estimation of the parameters was obtained using the maximum likelihood method (MLE), and assuming that the chance variables are independent and normally distributed. We considered a two-state disease model: cirrhosis and hepatocellular carcinoma and the absorbent state, death, that is irreversible (Figure 1). State 1 is that of patients with a diagnosis of liver cirrhosis (codes ICD9cm 571.2 and 571.5); State 2 is that of patients with a diagnosis of hepatocellular carcinoma (codes ICD9cm 155.0 and 155.2), regardless of the concomitant presence of a diagnosis of cirrhosis; State 3, absorbent, is that of deceased subjects. The few cases of patients who underwent liver surgery, including transplant, were excluded (codes ICD9cm for procedures 50.21÷50.99). Supposing, therefore, that progression of cirrhosis to HCC is an irreversible procedure, but in any case taking into account the possibility of misclassification between the first and second state and vice versa, we estimated the following transition intensities (Q) and misclassification (E) matrices:

The covariates inserted in the model were age, sex, the presence of disease correlated to alcohol abuse, the presence of diagnostic codes correlated to hepatitis C virus infection and the Charlson Index assessing the subject's clinical severity.

The Charlson index, developed in 1987 [19] and adapted to health data banks by Deyo et al. [20], is based on ICD9-CM diagnosis codes and contains 17 categories of comorbidity, each with an associated weight ranging from 1 to 6; the sum of all the weights gives the value of the index that, being determined in this way, takes into account both the number of comorbidities and their severity. The Charlson Index was divided into 2 classes: less than or equal to 3, greater than 3, as also was age: less than or equal to 65 years, older than 65 years. We selected 65 as the age cut-off because this is considered in the literature to be the mean age for development of liver cancer [21,22] and also because it corresponded to the median value of the observed distribution of cases of HCC, guaranteeing the stability of the model.

The analysis was conducting using the electronic Hospital Discharge Sheet coming from all the Apulia hospitals for the years 2000-2006, and Death Certificate DataBase related to all deaths events in Apulia for the years 2000-2006. From both databases were selected cases with the following ICD-9-CM (International Statistical Classification of Diseases and Related Health Problems 9^th^revision Clinical Modification) as principal or secondary diagnosis:

Liver cirrhosis (LC): 571.2; 571.5

Hepatocellular carcinoma (HCC): 155.0; 155.2.

To establish the starting point of the first state, defined as of the first hospitalization with LC diagnosis, were selected patients in whom the onset of cirrhosis presumably occurred between 01/01/2000 and 31/12/2000. For this purpose, we eliminated patients who had been hospitalized at least once for cirrhosis or hepatocellular carcinoma in the years 1998 and 1999. After identification the clinical course of the selected cirrhotic patients was reconstructed searching others hospitalizations with diagnosis of LC or HCC in HDS databases, or death correlated with LC or HCC in HDS and DCDB databases.

## Results

In total, 1925 patients were identified who had a presumed onset of cirrhosis in 2000 and had undergone at least one transition of state by 31/12/2006 (Table 1). In 33 patients, after a hospital admission for hepatocellular carcinoma, a subsequent hospitalization occurred with a diagnosis of only cirrhosis despite not having undergone surgery. These subjects were considered to have been misclassified. Table 2 shows the frequency of the covariates considered in the model. Estimates of the mean time of persistence in each state, calculated according to the procedure established by Jackson et al. [12,23], are reported in Table 3. We calculated the times of persistence also for the different levels of the covariates. The shortest time of persistence in state 1 (months = 24.99; CI =18.35-34.04) was observed in male subjects aged > 65 years, with a Charlson Index > 3 and free from diseases correlated to alcohol abuse or hepatitis C virus. The longest estimated time (months = 178.92; CI = 151.13-211.82) was observed in female subjects aged ≤ 65 years, with a Charlson Index ≤ 3, the presence of disease correlated to alcohol abuse and absence of hepatitis C virus infection. The shortest time of persistence in state 2 (months = 15.63; CI = 1.95-125.38) was demonstrated in male subjects aged > 65 years, with a Charlson Index > 3 and the presence of diseases correlated to alcohol abuse and hepatitis C virus infection, whereas the longest estimated time (months = 48.91; CI = 37.99-62.97) was found in male patients aged ≤ 65 years, with a Charlson Index ≤ 3, and absence of diseases correlated either to alcohol abuse or hepatitis C virus infection. For each covariate, the proportionality of hazards was verified using the Schoenfeld residuals method (Table 4). The overall test shows strong evidence of the proportionality of hazards and all the variables contribute to this proportionality.

**Table 1:** Summary of the number of transitions of state in the data set.

From:	To:			Lost to Follow-up	Total
			
	State 1	State 2	State 3		
State 1	922	393	610	-	1925
State 2	33*	124	154	82	393

*Patients certainly misclassified

**Table 2:** Frequency of the covariates at the start of follow-up.

Covariate	N. patients (% of 1925 cirrhotic patients)	
Sex (Male)	1107	(57,51%)

Age (>65 years)	1027	(53,35%)

Charlson Index (>3)	142	(7,38%)

Hepatitis B Virus (*070.20, 070.33*)	1	(0,05%)

Hepatitis C Virus *(070.41, 070.44, 070.51, 070.54)*	49	(2,55%)

Alcohol-correlated disease *(291.-, 303.0-, 303.9-, 305.0-, 357.5, 425.5, 535.3-, 790.3)*	61	(3,17%)

**Table 3:** Estimates of the mean permanency times in the transitory states.

	State 1	State 2
Estimate (months)	44.93	35.19
St. Error	2.47	2.97
Lower limit	40.33	50.05
Upper limit	29.82	41.52

**Table 4:** Proportionality Test based on Schoenfeld's residuals.

Variable	p-value
Sex	0.316
Age class	0.567
Hepatitis C	0.749
Alcohol	0.288
Charlson Index	0.468

Overall	0.729

The parameters estimated for the hidden Markov model are reported in Table 5. The estimated intensity matrix demonstrates that cirrhotic patients have twice the probability (0.0151/0.0071) of developing a liver cancer than of dying without developing a tumour. Moreover, the probability of death is four-fold higher in a subject with a liver cancer than in a subject with only cirrhosis (0.0284/0.0071).

**Table 5:** Parameters and standard errors estimated with the hidden Markov model.

Parameter	Results of model				
*Transition Intensities (with covariates set at their mean values)*					
^▲^	0.0151 (0.0012)				
^▲^	0.0071 (0.0006)				
^▲^	0.0284 (0.0024)				
					
*Probabilities of misclassification (with covariates set at their mean values)*					
	0.0237 (0.0040)				
	0.1408 (0.0329)				
					
Covariates					
	Sex	Age class	Charlson Index	Alcohol	HCV
	
	0.7961(0.1377)*	0.4362(0.1202)*	0.1858(0.2036)	-1.3280(0.8806)	-0.5330(0.4792)
	-0.0185(0.1662)	0.8667(0.1744)*	-0.1982(0.3543)	-0.2327(0.9073)	-0.4910(0.7100)
	-0.1628(0.1485)	0.3437(0.1365)*	0.5391(0.1652)*	0.2792(0.9727)	-0.0214(0.3018)

^▲^Instantaneous probability of transitions between the states ( between LC to HCC;  between LC to Death;  between HCC to Death)

* Significant at alpha = 0.05

(The  are the coefficients which weight the contribution of each variable on the probability of transition between states; the minus sign indicates that probabilities decrease if value of covariate increase, the plus sign indicates an increase in transition probabilities if value of covariate increase)

The estimated odds ratios are reported in Table 6.

**Table 6:** Estimated Odds Ratios for the covariates inserted in the hidden Markov model (95% confidence intervals in brackets).

	Transitions of State		
	
Covariate	State 1 -> State 2	State 1 -> State 3	State 2 -> State 3
Sex *(Ref.: female)*	2.2168(1.6923-2.9083)	0.9816(0.7087-1.3596)	0.8497(0.6351-1.1369)
Age class *(Ref.: Aged≤65)*	1.5469(1.2221-1.9581)	2.3791(1.6905-3.3482)	1.4102(1.0791-1.8429)
Hepatitis C *(Ref.: no HCV)*	0.5868(0.2294-1.5011)	0.6120(0.1522-2.4612)	0.9787(0.5417-1.7685)
Alcohol abuse *(Ref.: no alcohol)*	0.2649(0.0471-1.4884)	0.7923(0.1338-4.6909)	1.3221(0.1965-8.8968)
Charlson Index *(Ref. Charls. Ind.≤3)*	1.2041(0.8078-1.7948)	0.8202(0.4095-1.6429)	1.7144(1.2402-2.3700)

**Figure 2:**
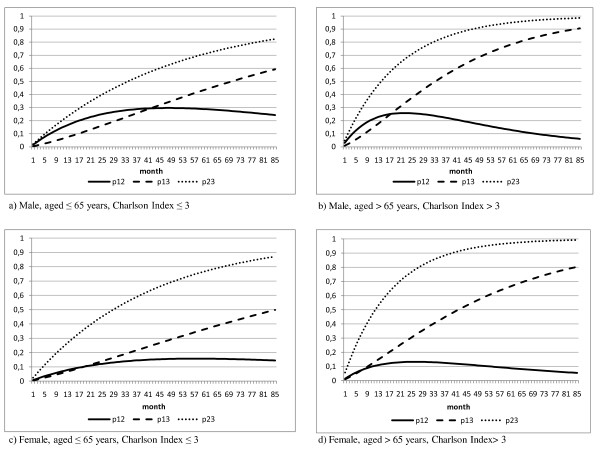
**Transition probabilities over time in cirrhotic subjects with no comorbidities correlated with alcohol abuse or hepatitis C virus**.

Figure 2 shows the trend over time of the estimated probabilities of transition in cirrhotic subjects free from comorbidities correlated to alcohol abuse or HCV infection. The probabilities of transition in Figures 2a and 2c are referred to cirrhotic subjects, male and female, aged 65 years or younger and with a Charlson Index of 3 or less.

The probabilities of transition from one state to the next are generally higher in males; in particular, the probability of progressing to HCC is never higher than 15.7% in females, whereas it reaches 30% in males after 4 years. In the first 40 months after the onset of liver cirrhosis, the probability of developing hepatocellular carcinoma is greater than the probability of death in males: 15.7% versus 6.8% after one year, 24.5% versus 15.5% after two years. In females, instead, the probability of death is little lower than that of developing HCC in the first few months, while already after two years the probability of death is higher (13.4% versus 11.8%).

In Figures 2b and 2d the transition probabilities are referred to males and females, respectively, with a more severe casemix (age > 65 years and Charlson Index >3).

The male-female differences in the probabilities of transition from cirrhosis to death grow over the first 3 years (being 10% at 36 months) but remain constant thereafter. The probability of developing HCC in the short term is higher in males than females (after 2 years, it is 25.8% in males versus 13.2% in females), but the values tend to converge in the longer term (6.3% in males versus 5.5% in females after 7 years).

The probabilities of death, both for subjects with only cirrhosis and with HCC, are generally higher than in subjects with a less severe casemix. In females the casemix does not seem to affect the probability of transition cirrhosis-HCC, whereas in males the more severe casemix has a long term effect when the probability of developing HCC declines in favor of a greater probability of death.

## Discussion

By exploiting the properties of Markov chains applied to a stochastic multistate model, we have calculated the temporal intensities of transition during the degenerative course of chronic liver cirrhosis. It was also possible to determine the time hazards of degeneration of the liver disease until death. Various studies have employed Cox models with the principal aim of determining the risk factors for the progression of cirrhosis to HCC [24,25]. In 2000, Degos et al. [26] studied progression to HCC and death using an "illness-disease-death" in a small cohort of subjects with a diagnosis of HCV-related cirrhosis, estimating the time of the events with the Kaplan Meier method. Then, in 2007 Ioannou et al. [27] applied the Cox proportional hazards model to a large administrative database of cirrhotic patients to determine the incidence of hepatocellular carcinoma, but without calculating the probabilities of transition from the first to the second state. Moreover, they acknowledged that a limit of their study was the risk of error when filling out the diagnosis codes ICD-9. In our study, using the data contained in the administrative health services databases, the multistate hidden Markov model we applied enabled us to identify the possible misclassification errors that can occur.

Previous studies based on surveillance programs for hepatocellular carcinoma in cirrhotic subjects reported an incidence of hepatocellular carcinoma of 1.5[28], 2.5[29], 6.7[30] per 100 subjects a year. In our study we found an incidence of HCC of 1.5% per year. Ioannou et al. (2007) [27], who conducted their analysis on an administrative database as we have done, found an incidence of 2.4% per year. The difference between these two values (1.5% vs 2.4%) is probably due to the different reference population, but also to insertion in our model of the misclassification matrix. In fact, the probability of misclassifying subjects with HCC as subjects with cirrhosis alone was revealed to be 14.08%, while the reverse error, i.e. misclassifying cirrhotic subjects as affected by HCC, was 2.73%.

The estimated parameters for the covariate "age class" were statistically significant for all the transitions. "Sex" was significant only for the transition of state from cirrhosis to HCC, while the Charlson Index had an effect only on the transition from HCC to death.

In various longitudinal studies it has been shown that advanced age and the male sex are associated with an increased risk of HCC in cirrhotic subjects [24,31-33], as was also shown in the present study. In fact, male cirrhotic subjects have approximately twice the probability of developing hepatocellular carcinoma as compared to female cirrhotic patients, while elderly patients (aged ≥ 65 years) have a higher risk of degeneration of the liver disease, and especially of dying while affected by cirrhosis.

The ample presence of concomitant diseases (Charlson Index ≥ 3) increases the risk of death in subjects with HCC. The Charlson Index was not found to have an incidence on the transition from cirrhosis to HCC. The insertion of specific comorbidities among the model covariates, such as diabetes mellitus [27,34,35], could help to identify the co-morbid conditions that may become risk factors for progression to HCC in cirrhotic subjects.

Alcohol is proposed to cause HCC mainly because it causes cirrhosis, whereas its association with HCC without cirrhosis is controversial and it probably has no direct carcinogenic role [36]. In a case-control study conducted in 2007, Kumar et al. [37] showed that although HCV RNA positivity and alcohol abuse significantly increased the risk of hepatocellular carcinoma among cirrhotic patients, no significant risk increase was evident in the absence of cirrhosis. In our study the estimates of the parameters related to hepatitis C virus and to the presence of morbidity correlated to alcohol abuse did not result significant. This result likely confirms that hepatitis C virus and alcohol abuse are risk factors for the onset of cirrhosis [38], but once the cirrhosis has become established they do not have an influence on the development of HCC.

Despite the advantages of the structured hidden Markov model approach, there are some limitations. As is typical of population dynamic models, collinearity between parameter estimates can lead to identifiability problems [39]. As a consequence, maximum likelihood estimates can sometimes yield implausible parameter values, and maximization algorithms may fail to converge [40]. The problem becomes particularly severe when time series are short and data scarce. Moreover, addition to the model of further covariates could exacerbate this problem. However, it seems likely that such problems with the proposed structured hidden Markov models might be overcome by adopting a Bayesian formulation.

## Conclusion

Markov model proved to be a useful tool for analysis of the course of a chronic degenerative disease like liver cirrhosis. In particular, the hidden Markov model that takes into account the risk of misclassification is well suited to the analysis of administrative health data because it can capture bias due to the problem that the data quality is not always optimal, as well as enabling the study of the effect of different covariates on the transitions of state.

A further improvement of the model could be that of predicting the state of subjects who have undergone surgery and appear cured or temporarily HCC-free. In our case, due to the paucity of such observations, it was not possible to consider a model like this.

## Competing interests

The authors declare that they have no competing interests.

## Authors' contributions

NB conceived the study, conducted the analysis, wrote the manuscript. PT conceived the study, collaborated in the analysis, drafted the manuscript. GS supervised the analysis and reviewed the manuscript. All authors read and approved the final manuscript.

## Pre-publication history

The pre-publication history for this paper can be accessed here:

http://www.biomedcentral.com/1471-2288/11/38/prepub
